# Music Streaming Services as Adjunct Therapies for Depression, Anxiety, and Bipolar Symptoms: Convergence of Digital Technologies, Mobile Apps, Emotions, and Global Mental Health

**DOI:** 10.3389/fpubh.2016.00217

**Published:** 2016-09-30

**Authors:** Karl Schriewer, Grzegorz Bulaj

**Affiliations:** ^1^Juan Diego Catholic High School, Draper, UT, USA; ^2^Skaggs Pharmacy Institute, College of Pharmacy, University of Utah, Salt Lake City, UT, USA

**Keywords:** wearable, medical device, telemedicine, antidepressant, randomized clinical trial, psychosocial, prevention, public health

## Introduction

Mobile technologies and music are recognized as opportunities to address mental health challenges (1–3), while clinical and economic benefits of mobile health (mHealth) are currently studied (4–6). Herein, we describe feasibility of repurposing music streaming services as therapies for affective disorders. According to the World Health Organization, there are 350 million people worldwide suffering from depression, and 60 million people living with bipolar disorder. Patients with affective disorders such as depression, anxiety, or bipolar spectrum, and their caregivers are challenged with managing disease symptoms, long-term treatments, and disabilities. Between 1990 and 2010, there has been a 41% increase in public health burden of mental, neurological, and substance use disorders, as measured by disability-adjusted life years (7). Depression accounts for 40.5% of total disability-adjusted life years among mental and substance-use disorders, whereas anxiety and bipolar disorder account for 14.6 and 7%, respectively (8). A long-term morbidity in bipolar spectrum disorders emphasizes the needs to improve treatments for depression (9). Treatments of affective disorders include mainly antidepressant, antipsychotic medications, and cognitive behavioral therapy (CBT). The efficacy of antidepressants for children and adolescent patients (10), medication adherence, and limited access to CBT in many countries continue to be a challenge for public health.

Treatments of depression, anxiety, and bipolar symptoms comprise CBT, psychosocial, and self-care interventions, also delivered *via* online and digital technologies (7, 11, 12). Opportunities for developing mobile apps and web-based interventions for neurological and mental disorders are economically feasible and coincide with the global adoption rates for smartphones (6, 13–16). Promising findings from clinical testing of mobile apps, e.g., in depression (17), are accompanied by challenges in patient engagement (18) and alignment of clinical and digital contents (19). Converting a mobile phone into low-cost virtual reality devices (exemplified by a Google VR cardboard) extends its potential medical applications (20, 21). Growing number of health-related wearables and devices measuring electrodermal activity (EDA), heart rate variability, or mobile electroencephalogram (EEG) systems, expand the use of digital technologies in medicine, including mental health [a 5-week treatment with EEG-based musical neurofeedback improved depression scores by 17% (22)]. Companies like Apple, Samsung, LG, Microsoft, Fitbit, Empatica, Emotiv, NeuroSky, or Muse develop smart watches and mobile EEG systems with health/wellness applications, whereas WellDoc, Akili Interactive, or Pear Therapeutics are engaged in converting mobile apps and games into medical device-based therapies for specific chronic diseases. Given accessibility of smartphones and the internet, we discuss opportunities for music streaming services to be developed as adjunct therapies and prevention of depressive, anxiety, and bipolar spectrum symptoms.

## Emotions and Music Streaming Services

**Figure 1:**
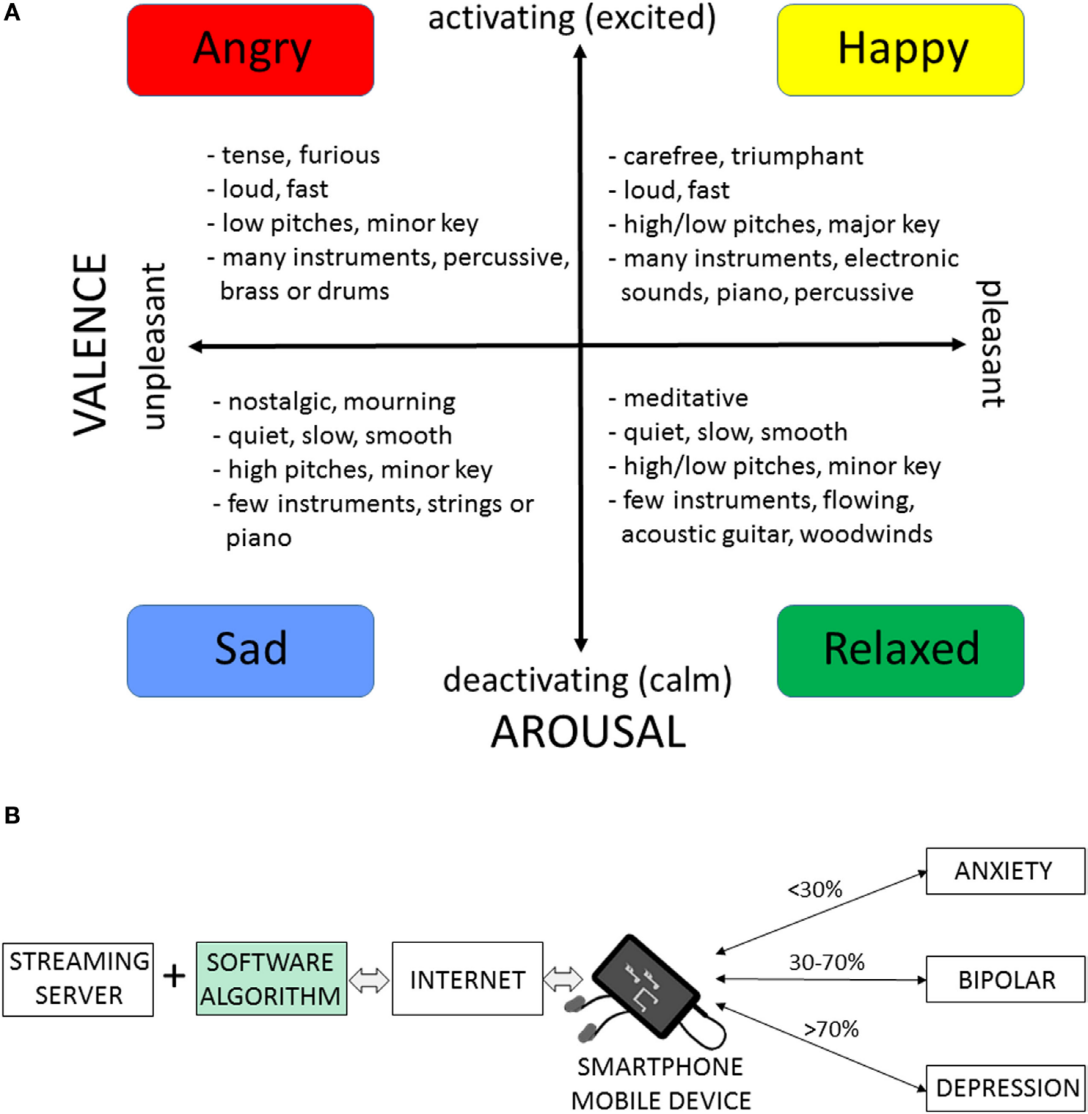
**Music streaming services as adjunct therapies for depression, anxiety, and bipolar symptoms by modulating arousal and valence**. **(A)** A valence-arousal plane illustrating types of emotion-related music. Characteristics in the four categories of music were selected from listening to mood-related music streaming services and searching for similarities in musical pieces classified by the music provider as similar within the same categories. **(B)** Schematic organization of a music streaming service as an adjunct therapy for patients with depression, anxiety, or bipolar spectrum disorders. Streaming server contains millions of musical tracks grouped into a wide selection of genres. Internet and mobile devices, such as smartphones, serve as the delivery system. After a listener selects the preferred type of music, the software algorithm generates a playlist for streaming clinically beneficial “dose” of music with positive valence and activating arousal. Hypothetical values express percent of time/songs with music activating arousal per day; these values can be determined for individual medical conditions (depression, bipolar, and anxiety). The streamed musical content is further optimized for target valence/arousal by interplay between the streaming software algorithm and the listener’s preferences and biofeedback (also contributing to outcomes and mitigating bias in clinical trials). Double-headed arrows emphasize the flow of music and the feedback mechanism. Rigorous testing of the clinical efficacy of music streaming software (green box) in randomized clinical trials is necessary to develop and validate the music streaming therapy.

Music modulates emotions by engaging several neurotransmitters and brain structures (2, 3, 23), including the brain’s reward and dopaminergic systems (24, 25). Music-induced emotions include happiness, relaxation, sadness, nostalgia, arousal, surprise, or irritation (Figure 1A). The relationships between musical structures and emotions are complex and can be described in terms of the BRECVEM mechanism (*Brain stem reflexes, rhythmic entrainment, evaluative conditioning, contagion, visual imagery, episodic memory*, and *musical expectancy*) (26). Music components such as tempo, dynamics, low/high pitches, satisfying rhythm, minor/major key, or instrumentation can change listeners’ arousal and valence, impacting her/his overall mood (Figure 1A). Music-induced emotional arousal and pleasure can be enhanced by expectation and predictability (27). Musical anticipation, specific rhythmic units, tempo, lyrics, and voice can affect arousal (28–30). Noteworthy, the musical harmony can contribute to intercultural differences in music perception (31). Specific musical structures such as those in the Mozart’s Sonata K.448 were found to activate parasympathetic nervous system and to reduce the frequency of seizures and epileptiform discharges in epilepsy patients (32), and these antiseizure effects were observed in pediatric and adult patients across western and eastern cultures. The rhythm of the K.448 piece appeared to be one of key “active” structures, as determined by comparing it with the retrograde version of K.448 (33). Positive emotional effects of music were measured using plasma oxytocin and vasopressin levels, pointing to connections between the neuroendocrine system, music, and emotions (34). Music can modulate emotional circuitry in patients with major depressive disorder (35) and music listening for at least 3 weeks can reduce depressive symptoms, with some randomized controlled studies reporting 19–47% improvements in depression scores (36, 37).

Music streaming services such as Amazon Prime Music, Apple Music, Google Play Music, iHeartRadio, Pandora, Spotify, or SoundCloud play a variety of songs on-demand *via* the Internet and according to the user’s interests and preferences. A person can select music stations based on specific songs, artists, genres, or mood of a song. The station then plays songs, which the listener already selected, as well as new songs, which are similar to previous songs played. Music streaming services offer millions of songs and musical tracks and are available worldwide, or in selected countries. Many stations offer mood-based categories, for example Spotify (“Have a Great Day,” “Mood Booster,” “Calm Down,” “Good Vibes,” etc.) and Apple Music (“Get Happy,” “100 Most Uplifting Songs Ever”) have preset playlists to choose, while Google Play Music (“Confident,” “Calm,” “Energetic,” etc.), Getty Images (“Up/Positive,” “Inspirational,” etc.), and Aupeo (“Happy,” “Dramatic,” “Relaxing,” etc.) have search criteria using mood to find specific music. Musicovery, another music streaming service, has a unique interactive interface similar to the arousal/valence plane (Figure 1A), in which the user can choose specifically what mood of music they would like. Music streaming services are compatible with Android and iOS operating platforms and include music video streaming like Vimeo or YouTube.

We hypothesize that digital and mobile technologies have advanced enough for repurposing music streaming service into a therapy for affective disorders by modulating arousal and valence *via* music-evoked emotions. While music streaming services have evolved to deliver on-demand music for entertainment purposes, many stations offer mood-based categories of music. It is unclear what criteria are used to choose specific pieces of music for particular mood-related categories, and they likely vary among music streaming services. Currently, there is a large diversity of mood-based stations, allowing a person to choose from, and listen to, a type of music that can affect her/his arousal (activating/deactivating) and valence (pleasant/unpleasant). As we describe below, before creating novel music libraries and streaming playlists for specific clinical purposes, individual pieces of music can be validated for their physiological responses using EDA and EEG systems.

Streaming music can modulate arousal and valence in people with affective disorders. For example, for patients with depression, who may otherwise prefer deactivating and negative-valence music (38), a long-term and daily stimulation with judiciously selected music (e.g., >60–70% of activating/arousal and positive/valence content) may produce additional clinical benefits. Similarly, for patients with anxiety, a daily streaming of calming/relaxing music can stabilize stress hormone levels regulated by the hypothalamic–pituitary–adrenal axis (2, 23). For people with bipolar disorder during the euthymic stage, a daily 30-min streaming of balanced activating/deactivating music with positive valence may help sustaining homeostasis of emotions and prevent relapses. Figure 1B illustrates how music streaming services could become adjunct therapies for patients with depression, anxiety, and bipolar spectrum disorders.

## Converting Music Streaming Services and Mobile Apps into Adjunct Therapies

Repurposing music streaming services into a medical treatment includes validation of clinical claims, followed by the regulatory clearance/approval for using software as a medical device (examples of the regulatory agencies include the European Medicines Agency or the Food and Drug Administration). In the United States, the Food and Drug Administration and the Federal Trade Commission ensure that marketing of digital health products is validated for specific conditions. Clinical validation of the music-generating software in randomized controlled trials is necessary to support the medical device status (Figure 1B). There are several mobile apps and games that have been cleared as medical device therapies for the patients with diabetes or stroke. To the best of our knowledge, there are no published data showing clinical efficacy of music streaming services for specific affective disorders.

To develop a music streaming service as an adjunct therapy for treatment of depression, anxiety, and bipolar spectrum, several parameters can be tested in randomized clinical trials, such as: (1) judicious selection of music with respect to arousal (activation/deactivation) and valence (pleasant/unpleasant), (2) total length of the therapy and daily duration of listening, and (3) proportion of arousal activating versus deactivating music delivered to patients daily and throughout the whole treatment. Such system is schematically illustrated in Figure 1B, and the key “medical” element is software with the approved medical device status. An affective brain–computer music interface provides an example of music-generating algorithms that could navigate affective trajectories for targeting desirable affective state during listening to music (39). Noteworthy, a mobile app delivering customized and downloaded music playlist for off-line use can be developed as mobile medical app. Coupling music with mobile EEG systems or EDA smartwatches offers additional means to increase the clinical efficacy of music-streaming by enhancing the physiologically active content through biofeedback mechanisms (22, 32, 39, 40).

Many patients with chronic medical conditions (diabetes, arthritis, neuropathic pain, schizophrenia, addiction, epilepsy, cardiovascular, cancer, and HIV/AIDS) who suffer from depression as comorbidity could also benefit from the music streaming therapies. Broader applications of music-evoked modulation of emotions *via* streaming include post-traumatic stress disorder, attention-deficit/hyperactivity disorder, autism, insomnia, neurorehabilitation, neurodegenerative, and developmental disorders. Since internet-based, self-help interventions may support prevention of depression (41), preventive medicine indications of music streaming may be also appealing for those at risk for affective disorders due to genetic, stress, adverse childhood experiences, and other environmental factors (42). Applications of music streaming can include postoperative pain management, given results from randomized trials on music interventions for analgesia in adult and pediatric patients (43, 44). When developing music-streaming medical software, protection of patient’s electronic health records, cybersecurity, long-term patient engagement (18), as well as unhealthy and maladaptive effects of music shall be taken into account (45, 46).

Increasing awareness among artists, music, digital, health care, and pharma industries about medical applications of streaming music could spur cross-disciplinary efforts toward development of low-cost, add-on therapies for chronic medical conditions. Given technological advances, biofeedback-based screening of already-existing musical tracks can facilitate initial evaluation of their clinical utility (47, 48). Analogous to drug discovery efforts using high-throughput assays, or virtual screening, collections of music can be mined with EEG, or EDA, or computer-based algorithms to detect and categorize specific musical structures. From the economic perspective, significantly higher revenues of pharmaceutical, electronic, and healthcare industries, as compared to copyright/music industry, favor mutual benefits of collaborative efforts to integrate creative elements into medical treatments. Challenges in advancing music streaming as medical treatments include: (1) differences between a rapid pace of technological advancements in consumer electronic industries and constant creation of new songs and music, as compared to a slower pace of clinical research and regulatory processes, (2) differences in business cultures between music/electronic industries and healthcare (profit margins versus the patient safety and clinical efficacy), and (3) individual and cultural preferences for different genres of music. Since music streaming services offer millions of musical tracks, and they constantly update their playlist portfolios, the above challenges could be mitigated by: (1) developing clinically effective, music-selecting algorithms that are independent of technological, or music content, updates, and (2) long-term collaborations between pharma and music streaming companies. Clinical validation and regulatory approval of music streaming has an incentive of broader acceptance by the patients, health-care providers, including potential reimbursements by third-party payers. Integration of music and mobile software with pharmacological treatments can lead toward development of molecular–behavioral combination therapies *via* drug–device combination products (14, 49). Such strategy also expands future portfolio of therapies, in addition to repurposing existing drugs, or developing new drugs, which can cost between $2 and $3 billion (50). Music streaming has a potential to improve health-related quality of life of people living with chronic medical conditions, further supporting public health.

## Conclusion

The diversity of cultural origins, music genres, and personal preferences does not impact universal values of music, while music therapies have been known for many years. Herein, we describe how a rapid growth in internet and mobile technologies, including worldwide accessibility of music streaming and smartphones can in part address increasing global mental health challenges. Repurposing music streaming services into the therapies for depression, anxiety, or bipolar spectrum will require cross-disciplinary collaborations and rigorous clinical validation of specific medical claims. Given convenience and low costs of delivering digital interventions, developing music streaming therapies could offer new opportunities for patients, their caregivers, health-care professionals, music industry, and artists worldwide.

## Author Contributions

GB conceived the project. KS analyzed and reviewed music streaming services and music structures shown in Figure 1. GB and KS reviewed literature, discussed the data, and wrote the manuscript.

## Conflict of Interest Statement

GB is a cofounder and the officer of Epicadence PBC, Public Benefit Corporation, a company developing music and mobile software as medical device therapy for epilepsy patients [this technology was previously described in Ref. (14)]. GB is also a co-inventor of patent-pending technologies “Disease Therapy Game Technology” and “Multimodal Epilepsy Management Suite.” In patent-pending application “Multimodal Epilepsy Management Suite,” there is a description of music streaming for epilepsy patients. KS declares no conflict of interest.
